# Seeking Clarity within Cloudy Effluents: Differentiating Fungal from Bacterial Peritonitis in Peritoneal Dialysis Patients

**DOI:** 10.1371/journal.pone.0028247

**Published:** 2011-12-01

**Authors:** Ruchir Chavada, Jen Kok, Sebastiaan van Hal, Sharon C-A. Chen

**Affiliations:** 1 Department of Microbiology and Infectious Diseases, Sydney South West Pathology Service, Liverpool Hospital, Liverpool, Australia; 2 Centre for Infectious Diseases and Microbiology Laboratory Services, Institute of Clinical Pathology and Medical Research, Westmead Hospital, Westmead, Australia; Los Angeles Biomedical Research Institute, United States of America

## Abstract

**Background:**

Fungal peritonitis is a serious complication of peritoneal dialysis (PD) therapy with the majority of patients ceasing PD permanently. The aims of this study were to identify risk factors and clinical associations that may discriminate between fungal from bacterial peritonitis.

**Methods:**

We retrospectively identified episodes of fungal peritonitis from 2001–2010 in PD patients at Liverpool and Westmead Hospitals (Australia). Fungal peritonitis cases were matched in a 1∶2 ratio with patients with bacterial peritonitis from each institution's dialysis registry, occurring closest in time to the fungal episode. Patient demographic, clinical and outcome data were obtained from the medical records.

**Results:**

Thirty-nine episodes of fungal peritonitis (rate of 0.02 episodes per patient-year of dialysis) were matched with 78 episodes of bacterial peritonitis. *Candida* species were the commonest pathogens (35/39; 90% episodes) with *Candida albicans* (37%), *Candida parapsilosis* (32%) and *Candida glabrata* (13%) the most frequently isolated species. Compared to bacterial peritonitis, fungal peritonitis patients had received PD for significantly longer (1133 vs. 775 catheter-days; p = 0.016), were more likely to have had previous episodes of bacterial peritonitis (51% vs. 10%; p = 0.01), and to have received prior antibacterial therapy (51% vs. 10%; p = 0.01). Patients with fungal peritonitis were less likely to have fever and abdominal pain on presentation, but had higher rates of PD catheter removal (79% vs. 22%; p<0.005), and permanent transfer to haemodialysis (87% vs. 24%; p<0.005). Hospital length of stay was significantly longer in patients with fungal peritonitis (26.1 days vs. 12.6 days; p = 0.017), but the all-cause 30-day mortality rate was similar in both groups. Fluconazole was a suitable empiric antifungal agent; with no *Candida* resistance detected.

**Conclusion:**

Prompt recognition of clinical risk factors, initiation of antifungal therapy and removal of PD catheters are key considerations in optimising outcomes.

## Introduction

Patients with end stage kidney disease (ESKD) undergoing dialysis are at increased risk of infections, with infection-related hospitalisation rates 31–68% higher than patients without kidney disease [1]. The route of dialysis determines the principal dialysis-associated infection type; peritonitis occurs almost exclusively in peritoneal dialysis (PD) patients. PD peritonitis is common (annual rates of ∼0.6 episodes per patient-year in the United States and Australia) [1], [2] and accounts for up to 16% of all deaths in PD patients [3]. Most peritonitis episodes are due to bacteria, with fungi causing only 2–10% of cases [4]–[6]. Although uncommon compared with bacterial peritonitis, fungal peritonitis is associated with higher mortality rates (5%–53% vs. 0.7%–15%) [4], [7] and often results in permanent discontinuation of PD with high morbidity [4], [8].

Management of PD peritonitis requires initiation of empiric antimicrobial therapy directed against Gram-positive and Gram-negative, organisms [3], whilst awaiting definitive identification and susceptibility data of the aetiologic pathogen. For fungal peritonitis, the guidelines also emphasise the importance of prompt catheter removal to reduce the high attendant morbidity and mortality [3]. Since laboratory results are often non-specific and mycological information may be delayed, it is important to recognise risk factors, clinical features and laboratory variables that discriminate fungal, from bacterial, peritonitis to guide early initiation of targeted antifungal therapy.

Herein, we examine clinical and epidemiological data from a multicentre case control study of PD patients to identify unique risk factors and clinical associations of fungal, compared to bacterial, peritonitis episodes.

## Results

A total of 1568 PD peritonitis episodes occurred in 2075 patients over the 9-year study period (rate of 1 episode every 15 patient-month or ∼0.8 episodes every patient-year). Thirty-nine (2.5%) episodes (in 39 patients) were due to fungi (rate of 0.02 episodes every patient-year). The median age of patients with fungal peritonitis was 64 years (range 51–77); 51% were female. Most episodes occurred in patients with their first PD catheter following mean PD duration of 1133 catheter-days (interquartile range 466–1710). All patients were receiving CAPD.

### Causative fungal pathogen

*Candida* species accounted for 35 of 39 (90%) fungal peritonitis episodes (Table 1). The remaining four episodes were caused by *Trichosporon beigelii*, *Rhodotorula mucilaginosa*, *Curvularia lunata* and *Prototheca wickerhamii* (one episode each). Thirty-three of fungal peritonitis episodes were monomicrobial, and six, polymicrobial (three episodes were caused by two different *Candida* species and in three other episodes, infection was caused by both a *Candida* and bacterium).

**Table 1:** Fungal isolates in thirty nine episodes of fungal peritonitis.

Fungal species	aNo. of isolates (%)
b ***Candida*** ** species (n = 38)**	
*Candida albicans*	14 (37%)
*Candida parapsilosis*	12 (32%)
*Candida glabrata*	5 (13%)
*Candida tropicalis*	3 (8%)
*Candida famata*	1 (2.5%)
*Candida guilliermondii*	1 (2.5%)
Non-speciated *Candida*	2 (5%)
**Non-** ***Candida*** ** species (n = 4)**	
*Trichosporon beigelii*	1
*Rhodotorula mucilaginosa*	1
*Curvularia lunata*	1
*Prototheca wickerhamii*	1

a Forty-two isolates were responsible for 39 episodes of fungal peritonitis; percentages are relative to total *Candida* isolates (n = 38).

b Thirty-eight *Candida* isolates (29 monomicrobial episodes with 29 *Candida* isolates and 6 polymicrobial episodes with 9 *Candida* isolates [3 dual *Candida* infections and 3 mixed *Candida*/bacterial infections]).

Thirty-eight *Candida* isolates were recovered. Fourteen of 38 (37%) of *Candida* isolates were *Candida albicans*; 12 (32%) were *Candida parapsilosis*; five (13%), *Candida glabrata*; and three (8%), *Candida tropicalis*. There was a single episode each due to *Candida famata* and *Candida guilliermondii*. Two *Candida* isolates were not speciated (Table 1). Blood cultures performed in 27 fungal episodes isolated *C. glabrata* once in a patient with *C. glabrata* peritonitis. Two patients had concurrent/prior exit site infections with the same fungus recovered in the preceding five and 12 days, respectively, of their peritonitis episodes.

*In vitro* antifungal susceptibility testing performed on 36 speciated *Candida* isolates using Sensititre YeastOne (TREK Diagnostic Systems, Inc., Cleveland, OH) and/or Etest (AB BIODISK, Solna, Sweeden), demonstrated fluconazole MICs of ≤4 µg/ml (n = 32), 8 µg/ml (n = 2) and 16 µg/ml (n = 2) [9]. Amphotericin MICs available for 36 isolates were 0.032 µg/ml (n = 1); 0.12 µg/ml (n = 4); 0.125 µg/ml (n = 3); 0.25 µg/ml (n = 8); 0.38 µg/ml (n = 1); 0.5 µg/ml (n = 13); and 1 µg/ml (n = 6). Caspofungin MICs available for 24 isolates were 0.03 µg/ml (n = 3); 0.06 µg/ml (n = 8); 0.12 µg/ml (n = 3); 0.25 µg/ml (n = 4); and 0.5 µg/ml (n = 6).

### Comparison of fungal and bacterial peritonitis

Patient demographics, major co-morbidities, risk factors, clinical features and laboratory parameters associated with fungal, and bacterial, peritonitis episodes are summarised in Table 2. Underlying co-morbidities and causes of ESKD were similar in both patient groups; the single patient with ESKD due to systemic lupus erythematosus had fungal peritonitis (Table 2). Episodes of fungal peritonitis were associated with significantly longer duration of PD (1133 vs. 775 catheter-days, p = 0.016), greater likelihood of previous bacterial peritonitis (51% vs. 10%, p = 0.01) and receipt of prior antibacterial agents (51% vs. 10%, p = 0.01). Conversely, patients with bacterial peritonitis were more likely to have been anaemic and suffer hyperparathyroidism, as reflected by more frequent use of erythropoiesis-stimulating (p<0.005), and calcimimetic agents (p = 0.02) respectively (Table 2).

**Table 2:** Comparison of patients with fungal versus bacterial peritonitis.

Characteristics	Fungal Peritonitisn = 39 (%)	Bacterial Peritonitis (n = 78 (%)	P value
Median age (years ± SD)	64±13	61±13	0.20
Female	20 (51)	36 (46)	0.74
Diabetes mellitus	18 (46)	41 (53)	0.64
Ischaemic heart disease	16 (38)	35 (46)	0.84
Solid organ tumour	5 (8)	4 (4)	0.76
**Aetiology ESKD**			
Diabetes mellitus	18(46)	41(53)	0.64
Chronic Glomerulonephritis	7(18)	14(18)	0.79
Hypertension	5(13)	12(15)	0.80
Polycystic kidney disease	3 (8)	2(3)	0.33
Systemic lupus erythematosus	1	0	-
Other^a^	6(15)	9(12)	0.77
**Markers of ESKD severity**			
Anaemia	16 (41)	64 (82)	<0.005
Hyperparathyroidism	6 (15)	29 (37)	0.02
Duration of dialysis (catheter days)	1133	775	0.016
Prior bacterial peritonitis episodes	20 (51)	8 (10)	0.01
Receipt of antibacterial therapy within the preceding 30 days	20 (51)	8 (10)	0.01
**Clinical/Laboratory findings**			
Abdominal pain	33 (85)	77 (99)	0.04
Fever	10 (26)	42 (54)	<0.01
Hypotension	12 (31)	11 (14)	0.06
Median blood WCC (cells×10^9^/L)	12	10	0.05
Median effluent WCC (cells×10^6^/L)	2094	7973	0.16
Median CRP (mg/L)	190	179	0.73
**Outcomes at 30 days**			
Technique failure	34 (87)	19 (24)	<0.005
Infection related surgery	7 (18)	12 (15)	0.79
All-cause mortality	6 (15)	11 (14)	0.85

a Includes cancer chemotherapy-induced, analgesic nephropathy, chronic pyelonephritis, renal calculi disease. ESKD = end stage kidney disease.

Fever and abdominal pain were more common in bacterial compared to fungal peritonitis (54% vs. 26%, p<0.05 and 99% vs. 85%, p<0.05 respectively), while there was a trend towards fungal peritonitis if hypotension was present. Gram stain of peritoneal fluid showed the presence of yeast in 8/25 (32%) episodes; in 15 cases, no organisms were seen and in two, only bacteria were visualised although cultures subsequently yielded both *Candida* and bacterial infection. Other laboratory parameters in fungal and bacterial peritonitis were in general, similar although the median peripheral blood white cell count was higher in fungal peritonitis (p = 0.05) (Table 2).

### Treatment and outcomes

Fluconazole was the main antifungal agent prescribed in fungal peritonitis (33 or 82% of cases); six patients received intravenous fluconazole monotherapy for the duration of the treatment course whilst 27 received intravenous, followed by oral therapy. Liposomal amphotericin B monotherapy was given in 11% of episodes (one each of *R. mucilaginosa*, *P. wickerhamii*, *C. glabrata* and *C. parapsilosis* peritonitis). Voriconazole (oral) and caspofungin (intravenous) at recommended doses were given in one instance each of *C. lunata* and *C. tropicalis/C. glabrata* infection, respectively. Flucytosine was not prescribed. Patients with fungal peritonitis received antifungal therapy for a mean duration of 24 days (vs. 11 days of antibacterial therapy in bacterial peritonitis episodes; p = 0.017). The majority of fungal peritonitis patients were treated in hospital and had a mean length of stay of 26.1 days compared with 12.6 days for bacterial infections (p = 0.017). Fungal episodes were also associated with higher rates of PD catheter removal (79% vs. 22%, p<0.005) and permanent cessation of PD (87% vs. 24%, p<0.005); reasons for catheter retainment were death (n = 3 patients), refusal of removal (n = 2) and decision for palliative treatment (n = 3).

The all-cause 30-day mortality was similar in both peritonitis groups (15% vs. 14%, p = 0.853), as was the need for infection-related surgery (these included adhesiolysis, colectomy post perforation and resection with primary anastomosis) during catheter removal (18% vs. 15%, p = 0.79; Table 2). By univariate analysis, factors associated with increased mortality in patients with fungal peritonitis were age >65 years (p = 0.009), previous bacterial peritonitis (p = 0.02) and polymicrobial infection (p = 0.011) (Table 3). Conversely, PD catheter removal was associated with increased survival (p = 0.013); 67% (4/6) of deaths occurred in patients with persistent sepsis from retained catheters.

**Table 3:** Variables associated with mortality in patients with fungal peritonitis.

Variables	Diedn = 6 (%)	Survivedn = 33 (%)	P value
Univariate analysis			
Sex (Female)	3 (50)	17 (52)	0.09
Mean age (years ± SD)	77±5	62±13	0.01
Diabetes mellitus	3 (50)	15 (45)	0.83
Anaemia	4 (67)	22 (67)	0.34
Hyperparathyroidism	1 (17)	5 (15)	0.92
Previous peritonitis	6 (100)	14 (42)	0.02
Peritoneal dialysis catheter removed	2 (33)	29 (88)	0.01
Polymicrobial infection	3 (50)	3 (9)	0.01

## Discussion

Fungal peritonitis in PD patients remains an important clinical entity with significant morbidity and implications for the continuation of PD. In contrast to bacterial peritonitis, the majority of patients with fungal peritonitis cease PD permanently and commence haemodialysis. Heightened awareness and early recognition of fungal peritonitis is central to optimise patient care as well as preserving the existing renal replacement therapy option. In the present study, we have identified (relative to bacterial infection) a number of clinical variables that may assist clinicians in differentiating fungal from bacterial infection.

The proportion of peritonitis episodes caused by fungi in our study (2.5%) is lower than that reported in a previous Australian study (4.5%) [4]; however, this proportion may vary across different regions in Australia (0–15%), with the highest rates reported in indigenous populations in Western Australia (WA) and the Northern Territory (NT) [4]. The proportion of fungal peritonitis that was observed in our largely non-indigenous population of metropolitan New South Wales was similar to the rate reported in Australian states other than WA and NT (0–3.5%) [4]. Overall, the rate of fungal peritonitis (0.02 episodes per patient-year) was also lower than that previously observed (0.03 episodes per patient-year) [4].

A key finding of the present study was that a relatively prolonged duration of PD was significantly associated with increased risk for developing fungal peritonitis, an observation not previously reported. Fungal contamination of peritoneal fluid may result from inadequate sterile technique when connecting PD catheters to dialysate bags, exit site and catheter tunnel infections, intestinal perforation or fistulae track formation [10]. However, the relative protracted timeframe in which fungal peritonitis occurred in our study argues against inadequate sterile technique, particularly in the immediate period following commencement of PD. We postulate that this association may be in fact due to the late recognition of fungal peritonitis or because of the high rates of PD catheter retention following previous bacterial peritonitis episodes.

Diabetes mellitus was not a risk factor for fungal peritonitis in the present study, as have been previously noted [11], [12]. Consistent with the findings of others, prior bacterial peritonitis and receipt of antibacterial therapy in the preceding 30 days were associated with the development of fungal peritonitis [8], [13]. Fungal peritonitis may have been initially misdiagnosed as bacterial peritonitis prior to laboratory confirmation of the former, prompting the prescription of empirical antibacterial therapy. On the other hand, the change in intestinal flora as a result of antibacterial therapy promotes the proliferation and transmural migration of fungi across the intestinal mucosa, increasing the risk of fungal peritonitis in the peritoneal cavity [6], [8], [14]. Previous reports have noted that secondary fungal peritonitis can be prevented by prophylactic antifungal use (fluconazole or nystatin) in patients receiving antibacterial therapy [15], [16], but this is not a strategy that is widely adopted in PD centres within Australia [4], including ours. The benefits of antifungal prophylaxis have not been clearly defined [3] and could lead to emergence of more antifungal-resistant species as previously noted in other populations [3], [17]. In contrast to bacterial peritonitis, anaemia and hyperparathyroidism were not significantly associated with fungal peritonitis (Table 1) consistent with previous studies [18], [19]. However, we were unable to examine the potential effects of varying dosing regimens of erythropoeitin-stimulating agents with either bacterial or fungal peritonitis; this remains to be studied in future surveys.

Although observational studies have suggested that the clinical signs in fungal peritonitis are similar to those of bacterial infection [8], few studies have systematically compared the clinical presentation in these two entities. We observed that whilst abdominal pain is almost universal in bacterial peritonitis, the relative absence of abdominal pain and fever despite a cloudy effluent raises the greater likelihood of fungal peritonitis. Previous reports have documented fever and abdominal pain in 36% and 68% of patients with fungal peritonitis respectively [6] (cf. with 26% and 85% in the present study, Table 2). Sensitivity of gram stain is low in fungal peritonitis [3] due to the relatively large volume of fluid examined and presumably low fungal load; we visualised yeasts in only a third of instances in our study. Yet Gram stain of peritoneal fluid is recommended by the International Society for Peritoneal Dialysis in the management of PD peritonitis since the visualisation of yeasts allows prompt initiation of antifungal therapy [3].

In the present study, *Candida* species were the commonest cause of fungal peritonitis, with *C. albicans*, *C. parapsilosis* and *C. glabrata* the most often isolated (37%, 32% and 13% of *Candida* isolates respectively). Our data is similar to that of other studies where non-*albicans Candida* species were more common than *C. albicans* [4], [6], [8]. Guidelines recommend systemic amphotericin and flucytosine as empiric antifungal therapy whilst awaiting identification and susceptibility of the aetiologic fungus [3]. Given that no *Candida* isolates were resistant to fluconazole in our study, this azole agent is an appropriate alternate empiric antifungal agent, pending species identification and susceptibility. In one large series, fluconazole monotherapy was used in 90% of fungal peritonitis cases; however, the impact of this strategy is uncertain as microbiological data on causative species and susceptibility were not presented [4]. An echinocandin (e.g. caspofungin) may be required to treat azole-resistant yeasts such as *C. krusei* or *C. glabrata* peritonitis [9]. Hence, where possible, all *Candida* isolates should be identified to species level, and antifungal susceptibility testing done to guide therapy. Although non-yeast fungi are occasionally reported to cause fungal peritonitis, *Candida* species still account for the majority of cases [4], [6], [8]. The newer azoles, voriconazole and posaconazole may be alternatives to amphotericin B when filamentous fungi are cultured [3]. Systematic study of larger numbers of patients are required to determine their position in the treatment of fungal peritonitis.

The overall mortality rate of fungal peritonitis has been reported to be 9–60%; with increased age, abdominal pain (with or without fever) and retained PD catheters the most frequently identified factors predicting mortality [4], [5], [8], [20]. In the present study, increased mortality was noted in those with retained catheters, emphasising the importance of prompt catheter removal when managing fungal peritonitis [3]. Although late (especially after 5 days following diagnosis) catheter removal has been associated with poorer survival [6], [21], this was not found in this and several other studies, perhaps due to the high rates of catheter removal at the outset and prompt initiation of antifungal therapy [4], [22]. This may have also accounted for the equivalent mortality rates between fungal and bacterial peritonitis that was observed, in contrast to previous reports of higher mortality rates for fungal peritonitis [3], [6]. Notably, fungal peritonitis patients with polymicrobial infection were more likely to die (Table 3). Fungal peritonitis was significantly associated with longer hospital stay, removal of the PD catheter, commencement of haemodialysis and receipt of systemic antifungal therapy.

The limitations of our study include the retrospective nature of the study, the lack of PD submodality analysis (continuous ambulatory vs. automated) as other potential risk factors for fungal peritonitis, and the potential biases that may have been introduced when matching fungal to bacterial peritonitis using PD registries, and by survivor bias. Our small sample size, although not enabling multivariate analysis for risk of death and involving only two large referral institutions, is nevertheless representative of the population of patients that suffer PD-related infectious complications.

In conclusion, fungal peritonitis is a serious PD-related complication. Morbidity is high and permanent cessation of peritoneal dialysis common despite adherence to management guidelines. Prompt recognition, especially in patients who have had their catheter *in situ* for a prolonged period, and removal of the catheter are key considerations in optimising outcomes.

## Methods

### Ethics Statement

Approval was obtained from the respective Human Research Ethics Committees of the Sydney West (SCA2008/8/5.3 [2846]) and Sydney South West (QA2009/071) Area Health Services. As the study was performed retrospectively with no intervention arm, the need for patient consent was waived by the Ethics Committees.

Episodes of fungal peritonitis occurring between January 2001 to March 2010 in PD patients at Liverpool and Westmead Hospitals (Sydney, New South Wales, Australia) were retrospectively identified from both institution's microbiology laboratory information systems and medical records. An episode of peritonitis was defined as the presence of any clinical sign of peritonism (including fever, abdominal tenderness and rebound tenderness) or peritoneal fluid (effluent) leucocyte count of >100×10^6^/L with a positive microbiological culture [4].

Fungal peritonitis cases were matched in a 1∶2 ratio with patients diagnosed with bacterial peritonitis from each institution's dialysis registry, occurring closest in time to the fungal episode. Denominator data obtained included the total patient-years of dialysis for the entire period.

Each patient's medical record was examined for demographic data and relevant history pertaining to ESKD (including aetiology, severity [as determined by erythropoietin-stimulating agent and phosphate binder/calcimimetic use], the date of PD commencement and duration of PD with the current catheter [inferred from the date of catheter insertion]). Data on previous peritonitis episodes, antibiotic use within the preceding 30 days, clinical symptoms and signs (e.g. fever, abdominal pain, hypotension) on presentation were also extracted, as was the presence of PD catheter exit site infection, antimicrobial therapy given, surgical procedures and hospital length of stay (if hospitalised). Laboratory data obtained include the cellular (white cells) profile results, identification and antimicrobial susceptibility profile of all positive peritoneal fluid and blood culture isolates, haematological and biochemical parameters, and C-reactive protein. Clinical outcomes including overall (all cause) 30-day mortality and technique failure (defined as permanent cessation of PD) were recorded.

Data were analysed using SSPS statistical software (version 18.0, IBM, Chicago, IL). For univariate analyses, categorical variables were analysed using Fisher's exact test or χ^2^ test and for continuous variables, the student's *t*-test; p values<0.05 were considered statistically significant. Multivariate analysis was not performed due to the small sample size of fungal peritonitis cases (n = 39).

## References

[1] U.S. Renal Data System (2010). http://www.usrds.org/default.asp.

[2] Australia and New Zealand Dialysis and Transplant Registry 33rd Annual Report 2010 (Data to 2009) (2010). http://www.anzdata.org.au/v1/report_2010.html.

[3] Li PK, Szeto CC, Piraino B, Bernardini J, Figueiredo AE (2010). Peritoneal dialysis-related infections recommendations: 2010 update.. Perit Dial Int.

[4] Miles R, Hawley CM, McDonald SP, Brown FG, Rosman JB (2009). Predictors and outcomes of fungal peritonitis in peritoneal dialysis patients.. Kidney Int.

[5] Troidle L, Gorban-Brennan N, Kliger A, Finkelstein FO (2003). Continuous peritoneal dialysis-associated peritonitis: A review and current concepts.. Semin Dial.

[6] Wang AY, Yu AW, Li PK, Lam PK, Leung CB (2000). Factors predicting outcome of fungal peritonitis in peritoneal dialysis: Analysis of a 9-year experience of fungal peritonitis in a single center.. Am J Kidney Dis.

[7] Mujais S (2006). Microbiology and outcomes of peritonitis in North America.. Kidney Int.

[8] Prasad N, Gupta A (2005). Fungal peritonitis in peritoneal dialysis patients.. Perit Dial Int.

[9] Clinical Laboratory Standards Institute (CLSI) (2010). Performance standards for antifungal susceptibility testing; sixteenth informational supplement..

[10] Gandhi BV, Bahadur MM, Dodeja H, Aggrwal V, Thamba A (2005). Systemic fungal infections in renal diseases.. J Postgrad Med.

[11] Matsuzkiewicz-Rowinska J (2009). Update on fungal peritonitis and its treatment.. Perit Dial Int.

[12] Ram R, Swarnalatha G, Neela P, Murty KV (2008). Fungal peritonitis in patients on continuous ambulatory peritoneal dialysis: a single-centre experience in India.. Nephron Clin Pract.

[13] Bren A (1998). Fungal peritonitis in patients on continuous ambulatory peritoneal dialysis: a report of 17 cases.. Eur J Clin Microbiol Infect Dis.

[14] Chen C, Ho M, Yu W, Wang J (2004). Fungal peritonitis in perioneal dialysis patients: effect of fluconazole treatment and use of the twin-bag disconnect system.. J Microb Immunol Infect.

[15] Restrepo C, Chacon J, Manjarres G (2010). Fungal peritonitis in peritoneal dialysis patients: Successful prophylaxis with fluconazole, as demonstrated by prospective randomized control trial.. Perit Dial Int.

[16] Wong P, Lo K, Tong GM, Chan SF, Lo MW (2007). Prevention of fungal peritonitis with nystatin prophylaxis in patients receiving CAPD.. Perit Dial Int.

[17] Pfaller MA, Diekema DJ (2010). Epidemiology of invasive candidiasis: a persistent public health problem.. Clin Microbiol Rev.

[18] Kestenbaum B, Sampson JN, Rudser KD, Patterson DJ, Seliger SL (2005). Serum phosphate levels and mortality risk among people with chronic kidney disease.. J Am Soc Nephrol.

[19] Mohanram A, Zhang Z, Shahinfar S, Keane WF, Brenner BM (2004). Anemia and end-stage renal disease in patients with type 2 diabetes and nephropahty.. Kidney Int.

[20] Oygar DD, Altiparmak MR, Murtezaoglu A, Yalin AS, Ataman R (2009). Fungal peritonitis in peritoneal dialysis: Risk factors and prognosis.. Renal Failure.

[21] Chang TI, Kim HW, Park JT, Lee DH, Lee JH (2011). Early catheter removal improves patient survival in peritoneal dialysis patients with fungal peritonitis: Results of ninety-four episodes of fungal peritonitis at a single centre.. Perit Dial Int.

[22] Wong P, Lo K, Tong GM, Chan SF, Lo MW (2008). Treatment of fungal peritonitis with a combination of intravenous amphotericin B and oral flucytosine, and delayed catheter replacement in continuous ambulatory peritoneal dialysis.. Perit Dial Int.

